# What shapes job satisfaction for middle nurse managers in hospitals and community care? A qualitative study

**DOI:** 10.3934/publichealth.2026043

**Published:** 2026-07-29

**Authors:** Dhurata Ivziku, Maria Pia Clasadonte, Idriz Sopjani, Gabriella Facchinetti, Marzia Lommi, Pasquale Iozzo, Fabrizio Polverini, Roberta Zanetti, Daniela Tartaglini

**Affiliations:** 1 Department of Health Professions, Fondazione Policlinico Universitario Campus Bio-Medico, Rome, Italy; 2 Department of Nursing, Faculty of Health Sciences, AAB College, Pristina, Kosovo; 3 Department of Health Professions, Local Health Authority Rome 1, Rome, Italy; 4 National Center for Clinical Governance and Excellence in Care, Istituto Superiore di Sanità, Rome, Italy; 5 Department of Biomedicine and Prevention, University of Rome Tor Vergata, Rome, Italy; 6 Department of Strategic Directorate, Local Health Authority 1 Liguria, Bussana di Sanremo, Italy; 7 Research Unit in Nursing Science, University Campus Bio-Medico, Rome, Italy

**Keywords:** middle nurse managers, job satisfaction, qualitative research, community healthcare, hospital nursing management

## Abstract

**Background:**

Middle nurse managers (MNMs) play a pivotal role in healthcare organizations, acting as a bridge between senior management and frontline staff. Despite their strategic importance, their job satisfaction remains insufficiently explored, particularly within the Italian healthcare context.

**Aim:**

To identify the organizational, relational, and professional sources of satisfaction and dissatisfaction characterizing the MNMs' role, seeking to capture the complexity of MNMs' experiences and to foreground their voices and narratives within the Italian healthcare context.

**Methods:**

A qualitative descriptive design was adopted. Twenty-five middle nurse managers were recruited using purposive sampling and participated in semi-structured interviews. Data were analyzed through inductive content analysis following Elo and Kyngäs' approach.

**Results:**

Four main categories emerged: (1) “Being an invisible manager”; (2) “Navigating communication across levels”; (3) “Working under pressure while finding meaning”; and (4) “Growing alone”. Participants reported limited formal and financial recognition, restricted involvement in decision-making, and challenges in work–life balance. Communication with senior management was often perceived as inadequate, particularly in hospital settings, whereas community-based managers described greater inclusion and appreciation. Despite scarce organizational support, many participants pursued self-directed professional development. Notably, the COVID-19 pandemic was described as a paradoxical period of increased professional fulfillment and team cohesion.

**Conclusions:**

Middle nurse managers' job satisfaction is strongly influenced by recognition, communication, working conditions, and access to professional development. Targeted organizational strategies aimed at enhancing recognition, leadership support, and structured training opportunities are needed to strengthen MNMs' well-being, retention, and contribution to healthcare quality.

## 1. Introduction

Job satisfaction, defined as a positive emotional state arising from job appraisal [1], is a key construct in organizational psychology [2] and an essential indicator of organizational health [3]. In healthcare—characterized by complexity and emotional demands [4]—satisfaction influences both staff well-being and care quality [5]. Its determinants include working conditions [6], organizational support, interpersonal relationships [7], recognition and autonomy [8], participation in decision-making, professional development [9], and leadership [10]. These determinants can be conceptually situated within a job demands–resources perspective, where leadership, recognition, autonomy, and professional development act as protective resources, while workload, role ambiguity, and limited decision-making authority represent key demands [11].

Conversely, dissatisfaction may lead to absenteeism [12], turnover [13], stress, and burnout [14], with negative implications for both workers and patient safety [15]. Middle nurse managers (MNMs), responsible for coordination and acting as a bridge between leadership and staff [16], play a pivotal role. Although job satisfaction has been widely examined in nursing research, the subjective experience of MNMs remains underexplored, particularly within the Italian healthcare system. Existing Italian studies have mainly focused on staff nurses or senior leadership roles and have relied predominantly on cross‑sectional quantitative designs [5],[13],[17] or psychometric validation studies [8],[10],[18]. While these contributions have advanced knowledge on leadership style, empowerment, and organizational outcomes, they provide limited insight into how MNMs perceive and make sense of their job satisfaction in everyday practice. Moreover, available evidence rarely addresses the organizational and contextual complexity of the MNM role, nor does it explore potential differences between hospital and community care settings. Qualitative investigations capturing MNMs' lived experiences, relational positioning, and role tensions within the Italian National Health Service are particularly scarce. This gap limits understanding of the contextual and experiential determinants of job satisfaction in a role that is strategically positioned between senior management and frontline staff.

To provide a conceptual lens for understanding job satisfaction among MNMs, this study is informed by the Job Demands–Resources (JD‑R) model. According to the JD‑R framework, employee well‑being and job satisfaction emerge from the dynamic balance between job demands—such as workload, role ambiguity, emotional labor, and organizational pressure—and job resources, including autonomy, recognition, leadership support, participation in decision‑making, and opportunities for professional development [11]. This model is particularly relevant for middle management roles in healthcare organizations, which are characterized by high job demands and limited formal authority [19]. Within this perspective, job satisfaction is not viewed as the result of single factors but as the outcome of the interplay between structural, relational, and organizational conditions.

Within healthcare organizations in Italy, MNMs play a central role in connecting senior leadership with frontline professionals. Their responsibilities typically include coordinating nursing teams, managing human and organizational resources, supporting quality and safety initiatives, and facilitating communication across organizational levels. In community and territorial healthcare settings, MNMs also contribute to the coordination of multidisciplinary services and the integration of health and social care. However, the formal definition and scope of this role may vary across regions and organizations.

This study addresses this gap by adopting a qualitative design to explore MNMs' perceptions of job satisfaction within a Local Health Authority in central Italy, across both hospital and community care settings and to identify the organizational, relational, and professional sources of satisfaction and dissatisfaction characterizing the MNMs role, seeking to capture the complexity of MNMs' experiences and to foreground their voices and narratives within the Italian healthcare context.

## 2. Materials and methods

### 2.1. Study design

The study used a qualitative descriptive design with inductive content analysis, grounded in Lincoln and Guba's [20] naturalistic inquiry, to explore middle nurse managers' perceptions of job satisfaction while bracketing researcher preconceptions. Methodological rigor and transparency were ensured by following COREQ guidelines.

### 2.2. Study setting and participants

Data were collected in a large public Local Health Authority (LHA) in central Italy, operating within the Italian National Health Service, serving about half of the metropolitan population across six municipalities. This LHA is characterized by a complex organizational structure and an integration of hospital and community care. It comprises 26 macro-structures, four hospitals, several day-hospital services, and six community districts, which differ in density, geography, and socio-demographic composition, providing a diverse study context.

The 26 macro-structures represent the core components of the LHA management hierarchy, including the strategic directorate, clinical and administrative departments, and six territorial districts. These structures are responsible for the governance, management, and coordination of healthcare and administrative activities. In particular, the Strategic Directorate is responsible for the overall strategic management of the organization, while department directorates oversee staffing decisions, economic balance, and the coordination of healthcare services such as prevention, mental health, and hospital care. Territorial districts, in turn, ensure the provision of healthcare services to defined geographic areas and municipalities, delivering a wide range of health and social care services outside hospital settings, with the aim of providing accessible, continuous, and patient-centered care within local communities.

From a managerial perspective, the organization follows a vertically structured hierarchical model articulated across three main levels: strategic, intermediate, and operational. Within the nursing management line, the strategic level is represented by the Directorate of Health Professions, which is responsible for the overall governance of nursing care. The intermediate level includes senior nurse managers and heads of departments or areas, who coordinate care processes and support the implementation of organizational strategies. The operational level consists of care units, where healthcare services are directly delivered.

Within this structure, MNMs are positioned at the interface between the intermediate and operational levels. They report to senior nursing management and are responsible for coordinating nursing and support staff, typically supervising teams of approximately 15–40 professionals, depending on the care setting. Their main responsibilities include human resource management, organization of daily clinical activities, ensuring quality and safety of care, and implementing organizational directives. In community and territorial healthcare settings, MNMs also contribute to the coordination of multidisciplinary services and the integration of health and social care. Therefore, MNMs perform a key bridging role between management and clinical practice, translating strategic directives into operational activities while also representing frontline staff needs within the organizational hierarchy.

A convenience and purposive sampling strategy was adopted. The study aimed to achieve maximum diversity to capture a wide range of perspectives and experiences. Specifically, diversity was sought in terms of age, gender, work experience, and work setting. The LHA employs a total of 70 MNMs, all of whom were invited to participate in the research. Participants were selected from both hospital facilities and community care districts, with an effort to include nurse managers with varying levels of seniority. Of these, 30 expressed initial interest, and at the time of the interviews, 25 MNMs agreed to participate and successfully completed their interviews. While this level of participation is consistent with qualitative descriptive methodology, which does not require statistical representativeness, we acknowledge that the findings may not capture the perspectives of those who did not participate. However, the sample included managers with extensive managerial experience, as well as those more recently appointed to the role. We also ensured diversity in the complexity of the services managed, including both high-intensity hospital wards and less complex community care services. Eligibility required at least one year in a managerial role, and interviews were conducted by an experienced qualitative researcher.

### 2.3. Data collection

Data were collected between February and May 2025 through semi-structured interviews [21] conducted outside working hours in hospital or district settings. Interviews were conducted by a nurse manager with extensive clinical, managerial, and qualitative research experience. The researcher was not connected with direct hierarchical or operational relationships to the participants to reduce biases in the data collection process. The researcher remained conscious of proper position throughout the interview process, making efforts to maintain a neutral stance and promote open dialogue. To further manage the researcher–participant relationship, reflective notes were maintained during and after the interviews, allowing for critical reflection on any potential influences of the researcher's background on the data collection. Additionally, a senior researcher supervised sessions to ensure that any unconscious biases or power imbalances were addressed, in line with best practices in qualitative research methodology [22].

Recruitment was direct, with informed consent and confidentiality guaranteed. Sessions lasted 30–45 minutes, were audio-recorded, transcribed verbatim within 48 hours, and continued until saturation occurred. In total, the interviews generated approximately 125 pages of verbatim transcribed data.

The interview guide was based on a literature review and the FIASO study [23]. The FIASO study is a national report aimed at exploring the role, competencies, and organizational positioning of middle healthcare managers within the Italian National Health Service. It highlights key findings related to job satisfaction, including the importance of role recognition, leadership support, participation in decision-making, and organizational context as critical determinants of satisfaction. These insights informed the development of the interview guide, particularly the questions addressing role recognition, decision-making involvement, and perceived organizational support. The interview guide included nine open-ended questions (Table 1) and socio-demographic data (ID, anonymized nickname, age, gender, education, managerial experience, care setting, staff supervised).

**Table 1.** Interview guide.

N	Interview question	Explored area
1	Do you feel that the role of middle management nurse is recognized and valued within your healthcare organization? Can you provide specific examples?	Role recognition and professional valorization
2	What are the most rewarding aspects of your work? Describe a situation in which you felt particularly satisfied with your role.	Job satisfaction and meaningful experiences
3	Are there specific challenges you face in your role as a middle management nurse? Can you describe a significant challenge and how you managed it?	Role-specific challenges and coping strategies
4	Do you feel that your voice and opinions are considered in organizational decision-making? Can you provide examples?	Participation in decision-making
5	How is communication and collaboration managed within the nursing team and with senior management? Are there areas that could be improved?	Team dynamics and interprofessional communication
6	What level of support and resources does the organization provide for your professional and personal development? Have any training programs been particularly enriching?	Organizational support and professional development
7	How has the current or recent work situation (e.g., the COVID-19 pandemic) affected your work and job satisfaction as a middle management nurse?	Impact of external factors on work and well-being
8	To what extent do you feel you have a work–life balance? What strategies do you use to maintain this balance?	Work–life balance and personal strategies
9	Do you have any suggestions or recommendations for improving working conditions for middle management nurses?	Recommendations for organizational improvement

### 2.4. Data analysis

The research team engaged in regular discussions to validate the coding framework and resolve any discrepancies. Data were analyzed through inductive content analysis adopting Elo & Kyngäs methodology [24]–[26]. In the initial open coding phase, two researchers independently engaged deeply with the transcripts, carefully reading and annotating them with descriptive headings to capture all relevant aspects of the content. In the subsequent categorization phase, the codes generated by both researchers were compared, discussed, and systematically grouped into broader generic categories. Through an abstraction process, these generic categories were further consolidated into overarching main categories. To enhance analytical rigor, a third researcher reviewed consistency against the transcripts. Consistent with qualitative descriptive methodology, which prioritizes interpretive consensus over quantitative measures of agreement [24], we used independent coding and a narrative consensus process rather than reporting numerical inter‑coder reliability statistics. The research team engaged in regular discussions to validate the coding framework and resolve any discrepancies. When code incompatibility or discrepancies arose between the coders, the disputed codes were discussed in a collaborative session between the primary researcher and another coder to reach a consensus. If necessary, the definition of the codes was refined to ensure clearer distinctions and consistency in application. This iterative discussion process and code refinement ensured that the coding framework was both reliable and reflective of the data [27].

Microsoft Excel was used as a tool to organize and manage the codebook, allowing for easy tracking of revisions, code frequencies, and thematic patterns. A short example of the code–general category–main category structure can be seen in Table 2, which includes illustrations of data extraction and classification for better clarity. This approach is consistent with best practices in qualitative research and aligns with methodological recommendations for ensuring rigor in data analysis [27].

**Table 2.** Description of the generation of the main and generic categories.

Main category	Description of main category	Generic category	Description of generic category	Code	Quote
Being an invisible manager	Perceptions of visibility, acknowledgment, and legitimacy of the managerial role	To be acknowledged as a managerial figure	Recognition of managerial competencies and authority	Limited recognition of the managerial role	“Sometimes it feels like you're expected to do and know everything—from admin to clinical, even psychological support—but that's not our role.” [A1].
		Lack of financial recognition for their role	Perceived economic undervaluation of responsibilities	Inadequate compensation	“The legal recognition of a professional profile comes through financial recognition. As long as that is missing, the role itself will not be acknowledged. We are the core of this organization—they should not undermine our role but give us the recognition we deserve.” [A7].
		Participation in decision-making processes	Exclusion from strategic and organizational choices	Limited involvement in decision-making	“We express our opinions, our knowledge, but decisions are always made over our heads. They are never ours... we don't have the tools to manage them or even to stop them or try to shape them differently.” [A2].

Navigating communication across levels	Relational dynamics with team members, superiors, and other professionals	Internal communication with the team	Building trust and engagement within the team	Team recognition and reliance	“The more informal approach is preferable [...] I always try to wait for colleagues and speak in person [...] or use messaging apps like WhatsApp. I also use written handovers in agendas, just to leave a trace.” [A1].
		Vertical communication with upper management	Relationship and interaction with senior leadership	Lack of recognition from management	“At the top level, contact is very, very limited... phone calls go unanswered, emails go unanswered... it's quite difficult.” [A2].
		Interprofessional relationships	Collaboration across professional roles	Boundaries between professions	“You find yourself having to break those patterns where you're just seen as a nurse trying to manage a care pathway that may also involve medical aspects. Often, the effort you're putting in or the goal you're trying to achieve is not properly acknowledged.” [A16]

Working under pressure while finding meaning	Experiences related to workload, environment, and emotional impact	Managing clinical/organizational challenges	Daily problem-solving and operational difficulties	Coping with resource limitations	“[...] everything surrounding our work is lacking—materials, equipment, tenders, repairs, technology—everything is missing.” [A6]
		Gratification from the team and from patients/service users	Recognition and appreciation from colleagues and patients	Patient recognition	“[...] when patients thank you... when they express appreciation, for instance by leaving positive feedback with the Public Relations Office (URP).” [A4]
		The COVID-19 paradox and the current work situation	Increased satisfaction during COVID-19 compared to the present	Paradoxical gratification	“[...] COVID was a stressful and intense period, but paradoxically, it came with more recognition; I felt more satisfied—it sounds strange, but that's how it was.” [A8]
		The delicate balance between work and personal life	Difficulty separating personal and professional spheres	Constant availability	“My private life is affected to some extent by my job, as there is always very little free time available, even on days off. [...] You can't really disconnect, not even on Sundays, because if something happens, I am the first to be contacted. Unlike in the hospital, there are no other points of reference in the community setting.” [A11]

Growing alone	Opportunities and barriers to professional growth	Lack of specific training pathways	Absence of structured and role-specific programs	Lack of institutional support	“At present, I don't see any strategies for development and support. Everything is left to one's personal drive for growth—postgraduate courses, master's degrees, and advanced training. There's no structured educational framework.” [A1]
		Self-directed learning and individual responsibility	Reliance on personal initiative for development	Individual responsibility	“Everything that has enriched me came from outside—personal, academic, or volunteer experiences. Nothing came from within the organization.” [A1]

### 2.5. Rigor and trustworthiness

Rigor was ensured through credibility, dependability, confirmability, and transferability [20],[25],[26]. Credibility was enhanced through reflexive discussions among the research team, peer debriefing, and field engagement to build rapport and obtain rich data. Dependability was supported by clear documentation of the data collection and analysis process, ensuring consistency through inter-coder agreement and a structured coding procedure. Confirmability was ensured with an audit trail of field notes and memos linking data to interpretations and ensuring that interpretations could be traced back to the participants' perspectives. Transferability was addressed through detailed descriptions of the context, sampling methods, inclusion criteria, participant characteristics, and the data collection process to enable readers to assess the relevance of the findings to other contexts. Moreover, detailed category descriptions supported by participant citations enhanced transferability by providing rich, context-specific data that allows readers to assess the applicability of the findings to other settings or populations. Participants were invited to engage in member checking to review the accuracy and relevance of the categories and codes in relation to their experiences. This allowed the validation of the findings, ensuring they accurately represented the participants' perspectives. Any discrepancies or additional insights provided by the participants were integrated into the final analysis, enhancing the credibility and confirmability of the study.

Participant recruitment and interviews continued until no new essential meanings (codes or subcategories) emerged in the analysis. Data saturation was reached at the 22nd interview. The team recognized, during the 22nd interview, the point at which the descriptions were sufficiently rich and complete to capture the essential structure of the phenomenon under investigation. Additionally, three more interviews were conducted to explore any further concepts. The richness of data remained consistent and no new information was retrieved, thus confirming data saturation [27],[28].

### 2.6. Theoretical framework

This study is grounded in the JD‑R framework, which conceptualizes job satisfaction as the outcome of the balance between job demands and job resources [11]. In this perspective, job demands refer to the organizational, relational, and role‑related aspects of work that require sustained effort, while job resources include factors such as recognition, autonomy, leadership support, participation in decision‑making, and opportunities for professional development. This framework is particularly suitable for examining MNMs' job satisfaction, as this role is characterized by high demands and limited formal authority, combined with strong relational and organizational expectations. Rather than attributing satisfaction to single determinants, the model allows for an integrated interpretation of how multiple demands and resources interact in shaping MNMs' work experiences across different care settings.

### 2.7. Ethical considerations

The study received formal approval from the Internal Review Board of the LHA (Protocol Number 4806–2025) and was conducted in accordance with the Declaration of Helsinki [29]. Participation was entirely voluntary, and all participants were informed about the study's purpose and their right to withdraw from the study at any time without consequences. Anonymity and confidentiality were strictly maintained. Audio files were used solely for analytical purposes and were deleted after transcription. Each participant was identified with an alphanumeric code. Access to data was restricted to the research team only. All data were handled in compliance with ethical standards for qualitative research.

## 3. Findings

The sample included 25 nurse managers from a large Local Health Authority in central Italy. Participants were 32% male and 68% female, with a mean age of 52.1 years (*SD* = 7.5) and 10.2 years of managerial experience (*SD* = 9.0). Nearly half (48%) held a master's degree; 56% worked in hospitals and 44% in community service. Detailed information is provided in Table 3.

**Table 3.** Description of the sample (*N* = 25).

**Variable**	***N***	**%**		
Sex				
Male	8	32		
Female	17	68		
Level of education				
Bachelor's Degree	13	52.0		
Master's Degree	12	48.0		
Work setting				
Hospital	14	56.0		
Community	11	44.0		

	***Min***	***Max***	***Mean***	***SD***

Age (years)	34	62	52.1	7.5
Management experience (years)	2	30	10.2	9.0
Staff managed	7	75	21.9	17.2

### 3.1. Content analysis

The analysis generated four main categories describing middle nurse managers' experiences of job satisfaction across organizational contexts. Main category development followed an inductive-interpretive process, moving from descriptive categories to conceptual labels grounded in participants' narratives. These main categories reflect the emotional and relational meanings attributed to the role, highlighting tensions between responsibility, recognition, and organizational support. The main categories were conceptualized as (1) “being an invisible manager”, (2) “navigating communication across levels”, (3) “working under pressure while finding meaning”, and (4) “growing alone”. These main categories align with key dimensions of job satisfaction identified in the literature and offer a more nuanced understanding of how they are experienced in practice.

### 3.2. Main category 1: Being an invisible manager

This main category encapsulates the perceptions of MNMs as having roles that are both undefined and undervalued within the organizational hierarchy. This invisibility is not only reflected in the lack of formal recognition and financial compensation but also identifies deeper issues related to role ambiguity, de-professionalization, and weakened managerial identity, which directly affect MNMs' job satisfaction. Participants expressed frustration over their roles, describing how they often felt sidelined, reduced to operational or emotional labor, and disconnected from the strategic leadership functions they were expected to perform. “We are not managers, and I don't see myself as an intermediary figure between top management and the staff” [A3].

#### 3.2.1. To be acknowledged as a managerial figure

A pervasive issue identified across participants was the lack of recognition of their managerial roles, which often left them feeling overlooked and diminished within the healthcare setting. This lack of acknowledgment, coupled with being assigned tasks beyond their scope of expertise, led to frustration and diminished job satisfaction.

Metaphors such as “secretary” highlight a symbolic downgrading of the role and reinforce a perception of a shift in identity and invisibility within the organizational hierarchy. “I feel like a simple administrative assistant... often I end up acting like a secretary.” [A3]. One participant highlighted how managerial invisibility was reinforced by organizational bypassing: “...when they completely bypass you, it means that your role is not recognized at all...” [A7]. Viewing oneself as a support figure rather than a strategic leader illustrates the de-professionalization of the role. This perception reflects a broader issue in nursing leadership, where managerial roles are increasingly associated with administrative support rather than leadership and decision-making responsibilities that are central to the role's identity. Such perceptions can severely affect job satisfaction.

Additionally, the findings suggest that MNMs are often positioned as operational problem-solvers rather than strategic leaders. A MNM stated: “...everything extra is assigned to the coordinator, even if it's completely unrelated to our professional profile.” [A2]. This participant's statement reflects the core tension between what is expected and what is feasible within the constraints of the managerial role. This reveals the growing burden of administrative tasks that detract from the core functions of leadership and strategy.

An MNM remarked: “...we're not managers... it feels more like being a buffer, or even a punching bag. I don't feel like a manager.” [A5]. These powerful metaphors, “feeling like a punching bag” and “being a buffer”, underscore the emotional strain of the role, where MNMs absorb the pressures of organizational demands without the recognition or authority to address the root causes. These expressions indicate that MNMs perceive themselves as emotional and operational burden bearers and are consistent with the emotional labor theories in leadership, where managers are expected to absorb stress and frustrations from both staff and upper management. This situation can lead to dissatisfaction and ultimately to burnout.

However, not all participants perceived a lack of recognition. One community-based MNM reported feeling “often recognized and highly appreciated” within the organization [A17], suggesting that experiences of professional recognition may vary across organizational contexts. This divergent perspective contrasts with the dominant pattern of perceived limited recognition and highlights that MNMs' sense of professional appreciation was not uniform but appeared to be shaped by the specific relational and organizational environment in which they worked.

#### 3.2.2. Lack of financial recognition for their role

Another critical issue identified by participants was the lack of financial recognition for their roles. Economic recognition was a central concern, with many participants expressing that their managerial responsibilities were undervalued financially. Financial recognition serves as a tangible indicator of an individual's professional value, and its absence can lead to feelings of marginalization. This lack of financial acknowledgment not only affected MNMs' job satisfaction but also hindered their sense of professional legitimacy and organizational importance. “...if they don't even recognize the economic value of the role, how can they possibly recognize its organizational importance?” [A4].

The notion that MNMs are “the core of this organization” reinforces the critical importance of their role, and the lack of recognition leads to dissatisfaction. “...the legal recognition of a professional profile comes through financial recognition. As long as that is missing, the role itself will not be acknowledged...” [A7]. “We are the core of this organization; they should not undermine our role but give us the recognition we deserve.” [24].

The lack of contractual implementation was also highlighted. “...the contractual rules are not being applied as they should be—we are not being recognized at all.” [A5]. This statement reveals the disconnect between formal agreements and their implementation; the failure to apply contractual rules related to compensation and role recognition leads to a sense of injustice. The absence of adequate compensation and contractual acknowledgment may undermine perceptions of organizational justice and professional legitimacy, ultimately reducing motivation, engagement, and job satisfaction.

#### 3.2.3. Participation in decision-making processes

This subcategory explores the degree to which MNMs are included or excluded from decision-making processes and how this exclusion relates to feelings of powerlessness, role identity, and job satisfaction. Hospital-based MNMs frequently reported being systematically excluded from decisions that directly impacted their role and daily operations, which contributed to frustration, diminished professional identity, and decreased job satisfaction.

A common description expressed by participants was the lack of involvement in key decision-making processes. MNMs often felt their expertise and input were disregarded, and decisions were made without their input or consideration. “We express our opinions, our knowledge, but decisions are always made over our heads. They are never ours... we don't have the tools to manage them or even to stop them or try to shape them differently...” [A2]. This quote highlights the powerlessness felt by MNMs when decisions are made without their involvement. Their lack of control over important decisions not only undermines their role but also affects their professional identity, as they are unable to influence outcomes or implement changes that could improve operations.

Another source of frustration for MNMs was their lack of participation in formal meetings where critical decisions were made. Many MNMs felt that they were merely passive observers rather than active contributors. The lack of decision-making authority within formal structures leads to feelings of disenfranchisement and dissatisfaction, as the opportunity to shape organizational direction is withheld. “There is no decision-making power during negotiation or decision-making meetings. Most of the time, you just endure the decisions—you don't participate.” [A6].

Some MNMs described resorting to assertive or even aggressive behaviors to gain recognition. A4 admitted: “...Only if I raise my voice and put it in writing. If I don't write, no one pays attention—as always...” [A4]. This response can be interpreted as a defensive or resistance strategy developed by MNMs in reaction to their exclusion from decision-making processes. The need to raise their voices and formalize requests in writing suggests a form of desperation to be heard and taken seriously. This behavior highlights the emotional labor involved in leadership roles, where MNMs may feel forced to adopt more confrontational strategies to secure even basic recognition. Such actions can ultimately contribute to job dissatisfaction.

In contrast to the experiences of exclusion described by many participants, some community-based MNMs reported greater involvement in organizational decisions and more collaborative relationships with local leadership. A10 emphasized, “In the territorial setting, the coordinator is the only figure present at the managerial level... They are seen as the captain of the whole operation.” [A10]. A13 added, “Both the medical and nursing directors are involved. We hold regular meetings, and when there's a difficulty—emerging or potential—we organize ourselves accordingly.” [A13]. Similarly, A18 reported being “actively involved by both nursing and medical leadership in organizational decisions” [A18]. These participants perceived themselves as active contributors rather than passive recipients of decisions, suggesting that opportunities for participation may vary according to organizational setting and local leadership practices. Greater involvement in decision-making appeared to reinforce participants' sense of professional value and managerial identity, while also contributing to higher job satisfaction.

Although many participants expressed frustration with the lack of formal recognition, some also highlighted experiences in which they felt valued, particularly by their teams. These more informal forms of recognition, rooted in daily practice and interpersonal relationships, contributed positively to their overall job satisfaction. The relational aspect of recognition, although not organizationally formalized, helped balance the structural and organizational deficiencies that many MNMs faced.

This dual aspect of recognition—formal versus informal—illustrates the complex nature of job satisfaction among MNMs. While structural factors such as participation in decision-making and formal recognition were significant contributors to dissatisfaction, positive interactions with team members provided a sense of value and connection.

### 3.3. Navigating communication across levels

Communication emerged as a central theme in this study, highlighting the role of MNMs as essential “mediators” within their organizations. MNMs are tasked with translating directives from upper management while maintaining cohesion within their teams. The results reveal both the strengths and challenges of communication across different levels, emphasizing how communication dynamics shape the MNM role, job satisfaction, and leadership effectiveness.

#### 3.3.1. Internal communication with the team

In terms of internal communication, MNMs generally reported positive experiences within their teams. Many found that communication flowed smoothly, supported by a variety of tools and strategies to ensure effective information exchange and cohesion. As participant A20 noted, “communication with the nursing team is continuous, smooth, and calm. We hold briefings in the morning before starting the workday.” [A20]. Structured communication within the team and regular briefings ensure that everyone is on the same page and ready for the day. The practice of holding daily briefings suggests that communication within the team is a proactive effort to maintain coordination and manage workflow, contributing to a more organized and supportive environment.

Informal communication also emerged as a crucial element of effective team interaction. Participant A1 described the preference for informal and flexible communication channels: “the more informal approach is preferable ... I always try to wait for colleagues and speak in person ... or use messaging apps like WhatsApp. I also use written handovers in agendas, just to leave a trace.” [A1]. This approach allows MNMs to maintain close connections with their team, adapting their communication style to foster trust and clarity. The use of messaging apps and face-to-face interactions demonstrates a leadership strategy that prioritizes accessibility and immediate feedback, ensuring that team members remain engaged and informed. These informal tools help bridge gaps in formal communication channels but also reflect a gap in structured organizational communication. Despite the effectiveness of these tools, they indicate that formal systems may be lacking or insufficient in some contexts.

However, communication challenges arose when MNMs were required to convey directives from upper management to their teams. As A14 explained, “...getting the group to understand and accept changes in our work is not easy, especially when dealing with old-school staff... with an old way of thinking. It's hard to bring in fresh air when they've been on that path for so many years...” [A14]. MNMs act as “translators” within the organizational hierarchy, mediating between strategic directives and frontline practice. This quote reflects the difficulty MNMs face when trying to implement changes or new directives within a workforce that may be resistant to change. The challenge of bridging generational gaps or ingrained practices further emphasizes the mediator role of MNMs, who must balance the demands of upper management with the realities of their teams. It also illustrates how MNMs often need to adapt their communication strategies to overcome resistance and ensure that change is accepted, a dynamic that can increase stress and reduce job satisfaction.

#### 3.3.2. Vertical communication with upper management

Communication with upper management differed across settings, with notable differences between hospital and community contexts. In hospital settings, MNMs frequently reported limited interaction with upper management, characterized by unanswered communications and few opportunities for dialogue, which led to feelings of isolation and uncertainty. Participants described “...phone calls go unanswered... emails as well” [A2], “...very few meetings or opportunities for discussion...” [A11]. Communication failures were also described through experiences of organizational exclusion. One participant reported that important staffing changes had been implemented without any prior discussion with the coordinator: “They decided to remove some healthcare assistants without even contacting me beforehand” [A16]. These comments suggest a communication breakdown between MNMs and upper management, contributing to feelings of powerlessness and a lack of support. The inability to engage in meaningful discussions with top management can hinder MNMs' ability to carry out their managerial roles effectively, ultimately diminishing their job satisfaction and effectiveness as leaders. This lack of vertical communication can also impede the flow of critical information, leading to inefficiencies and missed opportunities for collaboration.

In contrast, MNMs in community settings reported more frequent and direct communication with upper management, which facilitated a greater sense of inclusion and trust. As A13 shared, “we meet at least once a week” [A13]. A21 also described a “positive relationship with local leadership, characterized by regular dialogue and mutual trust” [A21]. Regular meetings in community settings appear to foster a more collaborative environment, where MNMs are better able to contribute to decision-making and feel more supported in their roles. This more open communication structure in community settings likely helps enhance MNMs' sense of recognition and leadership, suggesting that less hierarchical environments may promote better communication and job satisfaction.

However, even in community settings, variability in communication existed: “There's not much room for dialogue or discussion with the nursing line manager... on the other hand, with the medical or health directorate, absolutely yes” [A23]. While communication with senior management was frequently described as limited and fragmented, a small number of participants reported more positive experiences characterized by regular meetings, direct dialogue, and collaborative problem-solving. These divergent perspectives suggest that communication patterns are not uniform across settings and may be influenced by local organizational cultures and management structures. Nevertheless, even in more collaborative environments, inconsistent communication may still limit MNMs' involvement in organizational processes and decision-making.

Participants' suggestions for improvement, such as “greater listening”, “involvement”, and “clarity”, highlight the need for more inclusive organizational practices. The call for more “regular consultation”, “structured meetings”, and “transparent communication processes” points to a broader need for improvements in vertical communication to support MNMs in their leadership roles.

Overall, vertical communication and participation in decision-making appear closely interconnected. In line with the literature, limited communication may reduce managerial effectiveness and negatively affect both job satisfaction and clinical outcomes, whereas structured communication may enhance engagement, coordination, and quality of care.

#### 3.3.3. Interprofessional relationships

Effective collaboration and communication among professional groups, particularly between nurses and physicians, emerged as a critical area influencing MNMs' ability to lead effectively. In community settings, where more open communication and clearer role definitions existed, MNMs reported better coordination and teamwork. As A11 explained, “...from a collaboration perspective with the district, it works well with the medical component, less so with the nursing one...” [A11]. This suggests that MNMs in community settings may find it easier to collaborate with physicians than with other nurses, potentially due to more defined roles and responsibilities. This dynamic illustrates how organizational structure and interprofessional relationships can impact the effectiveness of care delivery and teamwork.

However, tensions between nurses and physicians were also noted. As A7 remarked, “The relationship between doctors and nurses...recognition is not very clear” [A7], pointing to rigid role boundaries. This lack of clarity in role recognition between these two professional groups can lead to conflicts and hinder collaborative efforts, affecting overall job satisfaction and care coordination. Similarly, A15 highlighted the difficulty of challenging traditional models: “Efforts to coordinate care pathways are not always acknowledged.” [A15]. These findings point to the persistent power dynamics within healthcare settings, where physician-centered hierarchies limit the autonomy and leadership of MNMs. This challenge to professional identity can undermine the ability of MNMs to lead effectively, leading to frustration and decreased job satisfaction.

Finally, A12 emphasized: “...The persistent perception of the nursing role as secondary...” [A12]. This sentiment reflects the ongoing challenges MNMs face in asserting their leadership and clinical expertise within a traditionally physician-dominated hierarchy. The lack of recognition and respect for the nursing role can negatively affect job satisfaction and team cohesion, as it undermines the authority of MNMs in clinical and organizational settings.

### 3.4. Main category 3: Working under pressure while finding meaning

This main category encapsulates the complex and often conflicting nature of the work environment experienced by MNMs. This category reflects the combination of demanding working conditions, pressure to manage clinical and organizational challenges, and the search for personal and professional meaning in their roles. The findings highlight how MNMs navigate these pressures, balancing feelings of frustration with moments of fulfillment and purpose.

#### 3.4.1. Managing clinical/organizational challenges

The working conditions faced by MNMs are characterized by constant clinical and organizational challenges. Many participants described satisfaction when they were able to resolve daily problems and bring teams together. As A8 noted, “When there are problems—and there always are—and I manage to get everyone to play the same tune, I'm happy because I tell myself I've done my job well.” [A8]. This sentiment reflects the sense of accomplishment that MNMs experience when they successfully address everyday challenges, particularly when it involves coordinating their team effectively. Similarly, A9 stated, “Definitely, when I manage to resolve problematic organizational situations, I feel satisfied.” [A9]. This quote highlights the rewarding nature of problem-solving in a leadership context, where resolving issues contributes to MNMs' sense of purpose and satisfaction in their roles.

However, despite these moments of gratification, many MNMs also reported ongoing frustration with resource shortages and structural obstacles that hindered their ability to perform effectively. A6 captured this frustration: “Everything surrounding our work is lacking—materials, equipment, tenders, repairs, technology—everything is missing.” [A6]. This reflects the chronic challenges that MNMs face in terms of inadequate resources, which create significant stress and pressure in their daily responsibilities. The persistent lack of support and resources contributes to role overload, where increasing responsibilities are not matched by the necessary tools or organizational support, heightening the risk of burnout. A19 further emphasized the feeling of being unsupported: “...we are left to fend for ourselves... every day we fight against windmills...” [A19]. This statement captures the overwhelming sense of isolation and frustration that arises when MNMs are tasked with managing without sufficient resources or support. The image of “fighting against windmills” reinforces the futility that can accompany daily efforts to navigate organizational and clinical challenges.

Additionally, some MNMs reported difficulties in driving change and innovation within their teams. As A1 stated, “The desire to change something by bringing in real-world insights and broadening perspectives, improving work methods, and introducing innovations...” [A1]. However, as A14 pointed out, overcoming resistance from long-serving staff is a significant challenge: “Making the team understand and accept the changes in our work hasn't been easy... especially when dealing with old-school staff... with an old way of thinking, so bringing in a bit of fresh air, doing things a bit more innovatively, isn't easy when they've been set in their ways for so many years...” [A14]. This challenge of implementing change, particularly among long-serving staff members, reveals the tension between the desire for innovation and the reality of entrenched practices.

These statements highlight the significant barriers to change within healthcare organizations, where MNMs must navigate both organizational constraints and staff resistance. This dynamic reflects the pressure MNMs face in trying to innovate and improve their work environment while managing the ongoing challenges of resource limitations and resistance to change.

#### 3.4.2. Gratification from the team and from patients/service users

Despite the many challenges, many MNMs identified relationships with their teams and patients as major sources of job satisfaction. A1 highlighted the fulfillment they derive from the recognition and trust of their team: “The recognition of my professionalism by the team I coordinate.... the nurse or healthcare assistant refers to me and awaits my opinion before making decisions, even in matters related to clinical care.” [A1]. This connection with the team provides a sense of professional validation and reinforces the MNM's role as a leader. It reflects the importance of interpersonal relationships and recognition in fostering job satisfaction. In contrast, some participants described strong professional gratification deriving from their relational role within community services. One participant stated that the coordinator became “a point of reference for users, nurses, and physicians” [A22], highlighting how continuity of relationships strengthened the sense of professional meaning and visibility. Similarly, A25 described recognition from service users as a major source of satisfaction, stating that “my satisfaction came from users recognizing these changes” [A15]. These relational dynamics fostered a sense of professional validation and reinforced the MNM's leadership role, underlining the importance of interpersonal recognition in promoting job satisfaction and professional fulfillment.

Additionally, relationships with patients contributed to deep satisfaction, as A6 explained: “If, at our age, we have continued to do this job and accepted positions that carry such responsibilities—which ultimately fall solely on us—it is solely because of our commitment to the patient.” [A6]. For many MNMs, their commitment to patients serves as a strong motivator, helping them find meaning in their work despite the ongoing challenges. Community-based MNMs valued recognition from service users and families. A10 shared, “I felt particularly fulfilled when I began to notice that, over the years, the user population started to acknowledge the improvements—that they recognized a ‘before and after’—as community-based structures tend to serve the same catchment population over time.” [A10]. This long-term relationship with service users provides a unique form of recognition that is deeply satisfying, as it acknowledges the MNM's efforts over time to improve care outcomes. These findings emphasize that interpersonal relationships (with team members or patients) serve as key sources of intrinsic job satisfaction for MNMs, helping them find meaning in their work even when formal organizational recognition is lacking.

#### 3.4.3. The COVID-19 paradox and the current work situation

A specific category addressing COVID-19 emerged because participants were explicitly asked about the pandemic's impact on their work and job satisfaction, which elicited reflections on both challenges and unexpected positive experiences. The COVID-19 pandemic emerged as a significant and paradoxical experience for MNMs. While the pandemic created a period of heightened professional satisfaction and purpose, it also intensified workload and emotional strain. As A12 noted, “During the pandemic... I had to support the nursing team and act as a kind of mental coach” [A12], while A3 referred to it as “a positive experience” [A3]. These statements highlight the pivotal role MNMs played during the crisis, providing emotional and professional support to their teams.

Participants also expressed that the pandemic fostered a sense of unity and recognition, which bolstered their professional identity and job satisfaction. As A9 explained, “an enormous dress rehearsal... a major test that strengthened our self-esteem and our sense of satisfaction” [A9].

However, this increased sense of purpose was not sustained beyond the pandemic. Many MNMs reported a decline in recognition and worsening working conditions in the post-pandemic period: “We are now worse off than before” [A8]; “today we're back to how things were before” [A17].

This contrast between the pandemic's temporary boost in job satisfaction and the subsequent decline highlights a paradox: while the crisis temporarily enhanced professional identity and collective efficacy, these effects were not sustained. The rapid loss of recognition post-pandemic may reflect the transient nature of crisis-driven appreciation, as organizational recognition and support typically return to pre-crisis levels.

#### 3.4.4. The delicate balance between work and personal life

Maintaining work–life balance emerged as a significant challenge, with MNMs describing constant availability and difficulty disconnecting from work. As A22 stated, “The coordinator never truly stops being a coordinator” [A22], while A5 emphasized that in critical care, “...there is simply no separation between work and personal life” [A5]. Similar dynamics were reported in community settings, where MNMs are often the only point of reference: “You can't really disconnect... if something happens, I am the first to be contacted” [A11].

Some participants described attempts to limit availability, such as “I force myself not to respond... even if I'm not always successful” [A20] or temporarily disconnecting: “...for 15 days I didn't read any work emails” [A13]. However, these strategies were not always effective, highlighting the persistent demand for availability and the difficulty of balancing personal time with professional responsibilities. Differently, some participants reported being able to establish clearer boundaries between work and personal life. As A18 explained, “I enjoy being here, but when I'm outside work, I enjoy being outside work”, suggesting that individual coping strategies may partially mitigate the impact of role demands. This perspective contrasts with the experiences of participants who described constant availability and an inability to disconnect from work, further illustrating the variability of work–life balance experiences across settings.

These findings suggest that, although individual coping strategies exist, their effectiveness is constrained by organizational demands and role expectations. Persistent work–life imbalance may generate ethical dilemmas and contribute to stress, burnout risk, and reduced job satisfaction.

### 3.5. Main category 4: Growing alone

This main category reflects widespread dissatisfaction among MNMs regarding the lack of structured training and professional development opportunities. The findings highlight how the absence of role-specific training pathways, coupled with a reliance on self-directed learning, impacts the professional growth and job satisfaction of MNMs. While MNMs demonstrate resilience and initiative in their personal development, the lack of institutional support for training creates a gap in the competencies needed for effective management and leadership.

#### 3.5.1. Lack of specific training pathways

Nearly all MNMs reported the absence of structured training programs tailored specifically to their managerial roles. This lack of formal training left many feeling unsupported in their professional development. As A1 noted, “There is no structured educational framework... everything is left to one's personal initiative” [A1]. This statement reflects the frustration of MNMs who, despite holding managerial responsibilities, are left to navigate their professional development on their own. The absence of a clear, formal training structure undermines the ability of MNMs to enhance their skills in a focused and systematic way. Similarly, A24 emphasized, “No one has trained us... there is a complete lack of it” [A24]. The lack of formal training opportunities further contributes to a sense of inadequacy, as MNMs feel unprepared to meet the demands of their roles.

Participants reported gaps in key competency areas critical to their effectiveness, including human resource management, conflict resolution, administrative processes, procurement, and leadership skills. As A21 pointed out, “The courses are always the same... there is no specific training for our role” [A21]. This statement highlights the mismatch between the available training programs and the unique needs of the MNM role.

While general management or leadership courses may be offered, these do not specifically address the nuances and challenges of nursing management, leading to a lack of preparation in key areas. A4 echoed this concern: “There are some interesting courses... but they are not accessible to coordinators” [A4]. While most participants described a lack of structured managerial training, a few acknowledged the existence of potentially valuable educational opportunities and reported more positive experiences. As A19 noted, “Here in the XX Department, many training initiatives are organized” [A19], suggesting that access to professional development opportunities may vary across organizational contexts. However, these opportunities were often perceived as difficult to access or insufficiently aligned with the specific needs of the MNM role. This suggests that dissatisfaction may derive not only from the absence of training but also from perceived inequities in access to professional development opportunities. The mismatch between the skills required for the MNM role and the training opportunities available contributed to feelings of professional stagnation and dissatisfaction, as many MNMs were expected to address complex managerial challenges without adequate institutional support.

#### 3.5.2. Self-directed learning and individual responsibility

In response to limited organizational support, many MNMs assumed responsibility for their own professional development. As A15 stated, “*It was entirely up to me... no one ever gave me anything*” [A15]. Although most participants described the absence of structured managerial training pathways, a few acknowledged the availability of potentially valuable educational opportunities, even if these were perceived as difficult to access or insufficiently tailored to the MNM role. These contrasting perspectives suggest that dissatisfaction may derive not only from the absence of training itself, but also from perceived inequities in accessibility and organizational investment in managerial development. This reflects a common sentiment among MNMs, where personal initiative becomes the primary driver of their professional growth. For many MNMs, self-directed learning became the only viable path to acquiring the necessary skills and knowledge. While this approach reflects personal autonomy and initiative, it also underscores the frustration and sense of isolation that comes with having to rely on self-guided development rather than receiving formal support from the organization with having to rely on self-guided development rather than receiving formal support from the organization.

Despite these challenges, many MNMs viewed self-directed learning not only as a burden but also as a demonstration of their professional commitment and autonomy. The ability to develop independently allows them to take control of their careers and growth, which can be a source of empowerment. This self-directed approach can enhance professional identity and engagement, as it allows MNMs to pursue knowledge and skills in areas that are most relevant to their work. However, the absence of formal organizational support for development is a significant barrier that can hinder job satisfaction and long-term career progression.

Table 4 provides a summary comparison of hospital-based and community-based MNMs perceptions. It highlights key differences in their perceptions, experiences, and challenges, illustrating how organizational context shapes managerial roles, responsibilities, and professional development opportunities.

**Table 4.** Comparison between hospital-based and community-based MNMs.

Main category	Hospital settings	Community settings
Being an invisible manager	Greater exclusion from decision-making; stronger role ambiguity; limited recognition from senior management	Greater managerial autonomy and visibility; stronger inclusion in local decision-making
Navigating communication across levels	Hierarchical and fragmented communication; limited accessibility of upper management	More direct and frequent communication with local leadership
Working under pressure while finding meaning	Higher organizational pressure and bureaucratic burden; limited resources	Greater continuity with patients and stronger perception of relational impact
Growing alone	Lack of structured managerial training and organizational support	Similar lack of formal pathways, but greater informal autonomy and local problem-solving

## 4. Discussion

The study explored the experiences of 25 middle nurse managers working in both hospital and community settings within a large public healthcare institution, focusing on their perceptions of job satisfaction. Interpreted through the JD‑R framework [11],[30], these perceptions can be understood as the result of a dynamic interplay between organizational demands and the availability of relational and structural resources across care settings.

The first main category, “Being an invisible manager”, highlights that MNMs often felt reduced to “support staff”, excluded from decisions, and burdened by role ambiguity, fostering frustration and demotivation. Conversely, community-based MNMs reported greater inclusion. Within a JD‑R perspective, limited recognition, role ambiguity, and exclusion from decision-making can be interpreted as key job demands that erode job satisfaction, while inclusion, autonomy, and acknowledgment function as critical job resources [30].

Recognition is essential for satisfaction, performance, and retention. Given the longstanding challenges of role confusion, heavy workload, and stress, there is an urgent need to ensure that nurse managers have a meaningful voice to act, with clear, structured opportunities to participate in decision-making and alignment with senior leadership priorities [31]. Organizations must recognize the importance of fostering a culture and climate of meaningful recognition for nursing leadership roles by affirming the value of their work and its outcomes for staff, patients, and the institution [31]. Recognition from senior management is positively associated with engagement, empowerment, and organizational commitment [32]. Leadership recognition also enhances motivation, performance, and organizational outcomes [33]. Clear role definitions and greater autonomy reduce stress and improve satisfaction [34], while involvement in decision-making fosters ownership and control [33]. Investing in structured recognition programs, leadership development initiatives, and shared governance frameworks is therefore essential to promote MNM job satisfaction, improve retention, and ultimately advance the quality and safety of healthcare delivery.

The hospital–community differences regarding greater MNMs involvement and satisfaction appears critical. Community services are typically organized in flatter, smaller-scale structures with shorter reporting lines, broader generalist roles, and closer proximity to local managers, which facilitates direct access to decision‑making areas. In these settings, MNMs tend to assume broader and more autonomous roles, participating directly in care pathway planning, interdisciplinary coordination, and continuity of care across the healthcare network. By contrast, hospitals are characterized by more complex bureaucracies and stronger medical dominance, with physicians occupying the apex of professional hierarchies and often bypassing managerial chains, which can limit nurse managers' influence over organizational priorities and resource allocation [35]. In addition, the nature of patient flow and service mandates differs. Community and primary care settings emphasize continuity, coordination, and the orientation toward long-term patient management, prevention, and socio-health integration, which may foster greater visibility of the nursing managerial role and more direct involvement in organizational and decision-making processes. On the other hand, acute hospitals focus on high‑throughput, technology‑intensive episodes in which physicians' decisions largely determine workflows [36]. These contextual differences in organizational structures, decision-making pathways, care flows, and professional power relations may help explain why community-based MNMs perceive greater involvement, autonomy, and job satisfaction. Our findings therefore suggest that organizational context plays a crucial role in shaping MNMs' experiences and should be more explicitly considered in future studies comparing hospital and community settings. Therefore, further research among those work settings is needed to support our findings.

In the second main category, “Navigating communication across levels”, communication and collaboration experiences varied by interlocutor. Communication with nursing staff was generally open and effective, supporting daily problem-solving and clinical responsiveness. Conversely, exchanges with top management were often problematic, marked by limited feedback and difficulty conveying operational needs. This gap hindered leadership effectiveness, organizational alignment, and job satisfaction. These communication difficulties appeared particularly evident among hospital-based MNMs, who described communication with senior management as more hierarchical, fragmented, and difficult to access. By contrast, several community-based MNMs reported more frequent and direct interactions with local management, although experiences were not uniformly positive across all territorial settings. This difference may reflect the shorter organizational lines, closer managerial proximity, and greater operational integration typically characterizing community services [35]. Our findings therefore suggest that organizational context significantly shapes not only communication patterns but also MNMs' perceptions of support, legitimacy, and professional visibility. This lack of accessible and consistent communication with top managers may also contribute to feelings of powerlessness, loneliness, and uncertainty among MNMs. Consistent with the literature on unsupportive work environments, these conditions may weaken psychological resilience, reduce leadership effectiveness, and increase emotional strain among MNMs [37]. According to the JD‑R framework, insufficient vertical communication and limited managerial support represent a depletion of job resources, increasing strain and undermining motivation and engagement among MNMs.

The literature supports these findings, indicating that nurse managers who receive support and empowerment from senior leadership are more likely to report higher levels of job satisfaction [31]. Clear and consistent internal communication not only facilitates efficient information exchange but also fosters mutual trust, team cohesion, and a shared sense of responsibility, ensuring that all members feel valued and understand their roles in achieving collective goals [38]. In healthcare settings, professional cohesion and mutual respect are recognized as critical determinants of job satisfaction, with positive, collaborative nurse–physician relationships essential for effective team functioning and operational efficiency [39]. Moreover, high-quality interpersonal relationships with superiors enhance MNMs' engagement and proactive behaviors [32]. Adopting transformational leadership practices at the senior level can further promote similar leadership behaviors among MNMs toward their own teams [40]. Strengthening vertical communication and structured dialogue with top management could enhance managerial effectiveness, MNM satisfaction, and organizational cohesion.

The main category, “Working under pressure while finding meaning”, encompasses the management of clinical challenges, the gratification derived from team collaboration and patient care, the experience of the COVID-19 period, and the complex balance between work and personal life. Managing critical situations was seen as demanding yet rewarding, with problem-solving providing professional gratification despite ongoing staffing and resource challenges. This process aligns with the concept of proactive coping, which reinforces self-esteem and resilience among managers [41]. From a JD‑R perspective, the coexistence of high demands and meaningful relational resources helps explain why MNMs may experience both strain and professional gratification within the same working conditions.

Another key source of gratification is the professional recognition received from the MNM's team, highlighting the importance of interpersonal relationships in fostering a sense of belonging and job satisfaction. This is particularly relevant in healthcare settings, where mutual support and collaboration are crucial [42]. Additionally, MNMs emphasized that their relationship with patients serves as a central motivation, helping them remain resilient even under difficult working conditions [39].

Work–life balance is a major challenge for MNMs, whose constant availability undermines satisfaction and well-being. Blurred boundaries between work and private life are a key stressor for frontline leaders [43]. Within the JD‑R framework, constant availability and role overload can be conceptualized as chronic job demands that, if not balanced by adequate organizational resources, increase the risk of exhaustion and reduce job satisfaction [11],[30]. To cope, many adopt proactive strategies—avoiding after-hours communication or delegating—which support psychological detachment and autonomy, both essential to prevent burnout and foster recovery [44]. However, our findings also suggest that these strategies were not equally feasible across contexts. Several hospital-based MNMs working in high-intensity settings described constant availability as an unavoidable ethical and professional responsibility, particularly in critical care environments where clinical and organizational issues required immediate intervention. Similarly, some community-based MNMs reported difficulties disconnecting from work due to the absence of alternative managerial references within territorial services. These findings highlight the limits of individual coping strategies when organizational demands and staffing pressures remain persistently high. Therefore, the individual coping mechanisms have diminishing returns when structural conditions, work design, and organizational culture remain unchanged. Even the most capable nurse managers, who employ advanced coping strategies, face limited managerial agency when autonomy is restricted, resources are scarce, and demands are unrelenting [45]. This highlights the need for systemic changes to support MNMs in maintaining work-life balance and job satisfaction.

MNMs described the COVID-19 pandemic as a period of strong fulfillment and heightened purpose. Crises such as COVID-19 can catalyze a shared social identity among workers, a sense of “we‑ness” and collective efficacy that strengthens commitment and perceived meaningfulness of work even under extreme conditions [46]. Studies in Italy showed that increased collective efficacy enhanced team commitment and job satisfaction [47] and reinforced professional identity and caregiving values [48]. Similarly, studies in organizational psychology suggest that when employees experience continuity of social identity and collective engagement during disruptive events, this continuity is associated with greater job satisfaction and reduced feelings of isolation [49]. From a social identity perspective, crisis contexts heighten the salience of professional identity and interdependence, amplifying both internal cohesion and resilience; this can temporarily elevate satisfaction by reinforcing the intrinsic value of the caring role and a shared sense of mission. Evidence indicates that collective responses to crises can even shape enduring narratives of professional role and identity growth long after the immediate emergency subsides [50]. Our findings also highlight the ambivalent nature of the pandemic experience. While many MNMs described the COVID-19 period as professionally meaningful and emotionally unifying, they simultaneously reported intense workloads, emotional exhaustion, and prolonged psychological pressure. This paradoxical duality— heightened collective purpose coexisting with increased vulnerability to burnout—can be understood through the lens of identity theory and crisis research: the very psychosocial mechanisms that enhance cohesion and satisfaction in the short term may contribute to resource depletion and strain over time as intense engagement persists without adequate recovery or support [46]. However, participants state that recognition from top management quickly faded post-pandemic, exposing MNMs to deteriorated conditions and reduced support. International evidence confirms that such short-lived recognition leads to renewed stress, burnout, and disengagement [39]. Sustained strategies that ensure recognition, supportive leadership, and better working conditions are essential to strengthen MNMs' self-esteem, decision-making, and retention [51]. Another explanation for this rapid disappearance of appreciation expressed by MNMs can be generated by a complex array of institutional-level factors, including economic pressures, ongoing structural reforms, and staff shortages that exacerbate the workload of healthcare managers [48]. These challenges often lead to fatigued senior management, whose limited resources and attention span may result in diminished recognition of the contributions made by MNMs. This dynamic highlights the need for further research to explore how these broader institutional conditions impact managerial recognition and job satisfaction.

Finally, the main category, “Growing alone”, reports a striking ambivalence: while participants report limited organizational support, time, and resources for formal learning, they simultaneously demonstrate strong professional agency, relying on self‑directed learning and informal peer networks to update their knowledge and skills. This main category reflects systemic features of Italian nursing education. In Italy, continuing professional development is formally regulated through the ECM system, which aims to support lifelong learning and competence retention for all health professionals, including nurses and nurse managers. However, participation often depends on individual initiative rather than structured organizational pathways [52]. Research on nursing managerial training programs in Italy indicates that while structured Master's level courses exist, organizational support for applying newly acquired managerial competencies in practice is often limited, with contextual and structural factors hindering effective implementation [53]. Formal managerial education for nurses thus remains an evolving and fragmented area. Broader workforce planning challenges—such as demographic shifts in the nursing workforce, regional disparities in nurse distribution, and persistent nursing shortages within the public healthcare service—further constrain time and resources for professional development, amplifying the need for individual initiative to maintain and expand competencies [54],[55]. Collectively, these Italy-specific structural and policy factors help explain why formal support for professional learning is perceived as limited and why strong personal agency emerges as a compensatory strategy among MNMs. This gap, well-documented in the literature [32], undermines job satisfaction and hinders professional growth [38]. In JD‑R terms, the lack of structured training pathways represents a critical absence of job resources that are essential for sustaining motivation, competence, and long-term engagement in managerial roles. Lacking structured support, many MNMs pursue self-directed learning, suggesting significant professional agency and commitment to their own development. Though this pursuit of growth showcases their self-efficacy and dedication, it demands significant personal investment, and the absence of formal recognition often leads to frustration, lower motivation, and reduced engagement [56]. While self-directed learning is essential for the continuous professional development of healthcare providers, its success hinges on creating an environment that supports it. Institutions must foster self-directed learning-friendly environments by providing protected learning time, access to digital tools, and mentorship programs to enhance the effectiveness of self-directed learning initiatives and ensure that professionals are equipped to thrive in their roles [57].

Interpreted through the JD‑R framework, our findings suggest that MNMs' job satisfaction is primarily sustained by relational and organizational resources—which are unevenly distributed across hospital and community care settings—and that their erosion contributes to frustration, disengagement, and role strain. Accordingly, enhancing MNMs' satisfaction, retention, and well‑being requires sustained organizational investment in tailored education and leadership development programs, alongside the promotion of a supportive and appreciative work culture that fosters healthy work environments [42].

## 5. Strengths and limitations

This study provides rare insights into middle nurse managers' job satisfaction in Italy. Qualitative design captured rich experiences, enhancing knowledge on this issue, with participant diversity supporting transferability. By adopting the JD‑R framework as a theoretical lens, the study offers an integrated interpretation of how organizational, relational, and professional factors operate as demands or resources shaping MNMs' experiences across hospital and community settings. The inclusion of both care contexts represents an additional strength, allowing for a comparative understanding of how contextual differences influence the balance between demands and resources in middle management roles.

The major limitation of this study is the single-institution setting, a Local Health Authority in Italy, which may limit the transferability of findings to other healthcare settings. The LHA structure and regional policies can influence healthcare practices, and these specific contexts may not be representative of different countries or healthcare systems, for example, private versus public contexts. As a result, the applicability of our findings to other settings should be considered with caution, particularly in regions with different organizational frameworks or healthcare priorities. Additionally, the voluntary nature of participation and the potential for social desirability bias may have influenced the results, particularly concerning participants' views on satisfaction and management practices. Those who chose to participate might have different perspectives from those who did not, potentially skewing the data toward more socially acceptable responses. Furthermore, while subjectivity is an inherent aspect of qualitative research, we took several measures to minimize its impact. This study included narrations only from 25 participants, and no detailed information was collected from non-participants. Consequently, the findings may not fully capture the perspectives of those who did not participate, and transferability should be considered with caution. Reflexivity, the involvement of multiple investigators, and the use of audit trails were employed throughout the study to enhance transparency and ensure a rigorous analysis process. These steps allowed us to address and manage the potential biases introduced by individual perspectives, thereby strengthening the trustworthiness of the findings. Additionally, participants were explicitly asked about the pandemic's impact on their work and job satisfaction. Although Question 7 was open-ended, its phrasing may have influenced responses, contributing to the prominence of pandemic-related positive experiences and shaping participants' reflections on their current work context. Despite this, the study offers useful evidence to guide strategies for MNMs' satisfaction, retention, and development.

## 6. Implications for research, policy, and practice

The findings emphasize the urgent need for policies that enhance MNMs' involvement in decision-making and foster more effective communication with senior management. These policies should focus on recognizing MNMs' managerial competencies and reinforcing their sense of organizational belonging. To maximize MNMs' effectiveness, interventions must formalize their role in both strategic and operational decision-making, for example, by ensuring their routine participation in executive-briefing sessions and dashboard reviews [58]. These sessions could present unit-level data, challenges, and opportunities, promoting informed decision-making. Additionally, interventions must prioritize an organizational culture shift that normalizes MNM participation in decision-making as a standard expectation, rather than an exception [58]. Furthermore, the establishment of MNM-inclusive strategic councils or governance committees with clearly defined decision rights (e.g., policy revisions, staffing norms, unit-level budgeting) is essential. Ensuring MNMs serve as co-chairs or equal participants will help translate policy into practice and facilitate meaningful involvement in organizational leadership. When policies provide MNMs with autonomy and the necessary resources, they are better equipped to enact impactful changes [59],[60].

At the practice level, to address the problems evidenced by MNM in the first two main categories (*Being an invisible manager* and *Navigating communication across levels*), healthcare institutions should prioritize investments in structured recognition systems, incentive programs, and targeted training in relational and leadership skills to enhance job satisfaction, motivation, and retention. Training initiatives should emphasize trust-building, ethical leadership, and relational governance, which are key to improving MNMs' influence and credibility with senior management [61]. Additionally, implementing targeted leadership development programs for MNMs is crucial to overcoming the *Growing alone* narrative. These programs should focus on fostering transparent, two-way communication with senior leaders, inclusive decision-making, conflict resolution, change management, resilience, and ethical leadership. Formal mentoring or coaching pairings between MNMs and senior executives can facilitate upward feedback, ensure alignment of unit goals with organizational strategy, and help navigate resource constraints [61],[62].

To support the self-directed learning pattern highlighted in our findings, institutions should create environments that foster professional growth by providing protected learning time, access to digital tools, and structured peer networks. Multimodal communication strategies must be implemented to enable MNMs to effectively disseminate decisions and gather frontline input through executive summaries, dashboards, and structured forums for staff feedback. Regular climate assessments should be conducted to monitor the ethical climate, perceived support, and MNMs' sense of belonging, with results used to continuously refine development plans for MNMs [62].

To further support career growth and address the ambivalence highlighted by the COVID-19 paradox, healthcare institutions should develop career ladders, residency/preceptorship enhancements, and recognition frameworks, which can reinforce professional identity, motivation, and job satisfaction during periods of crisis and high workload. MNMs should receive structured onboarding and transition support to facilitate a smoother integration into their roles, promoting role clarity and professional autonomy, particularly in settings where responsibilities differ, as found in this research, for example, between hospital and community contexts. Psychological safety and interprofessional collaboration programs are vital for reducing silos, enabling MNMs to confidently engage in governance and decision-making activities, thereby strengthening collective efficacy and resilience as predicted by crisis-driven identity theory [62]. Moreover, monitoring retention metrics, job satisfaction, organizational commitment, and governance engagement will help assess the effectiveness of interventions, which should be adjusted based on data and feedback. Additionally, regular climate assessments, through surveys and focus groups, should be conducted to gauge ethical climate, perceived organizational support, and MNMs' sense of belonging, ensuring that development strategies and resource allocation are tailored to reinforce professional motivation, autonomy, and sustained satisfaction across both hospital and community settings.

Further studies are warranted to examine the impact of MNMs' participation in decision-making on organizational climate and patient outcomes. Specifically, research should explore how increased MNM involvement in governance and policy formulation affects the overall work environment, staff morale, and quality of care provided. Additionally, longitudinal studies could assess the long-term effects of MNM participation on patient safety, clinical outcomes, and team performance. It would also be beneficial to evaluate the effectiveness of specific training and development programs designed to enhance leadership, communication, and team management skills in MNMs. Research could focus on which types of training—such as conflict resolution, change management, and ethical leadership—yield the most significant improvements in MNM effectiveness and job satisfaction. Comparative studies across different healthcare settings and regions could help identify best practices and strategies for maximizing the impact of leadership development programs. Furthermore, investigating the role of organizational culture in facilitating or hindering MNMs' participation in decision-making could provide valuable insights. Understanding the barriers and enablers to effective leadership at the MNM level would help tailor interventions to specific organizational contexts. Finally, exploring the interplay between policy environments, resource allocation, and MNM empowerment would be critical in designing systemic changes that support sustainable leadership models in healthcare.

## 7. Conclusions

MNMs' job satisfaction revolves around four main categories: “Being an invisible manager”, “navigating communication across levels”, “working under pressure while finding meaning”, and “growing alone”. These factors—already highlighted in literature—remain crucial for well-being and motivation. The study adds new insights, showing that the COVID-19 crisis temporarily boosted MNMs' fulfillment, motivation, and perceived value, and that community-based managers felt more recognized than hospital-based ones. Strengthening recognition and compensation, investing in targeted development, and ensuring regular engagement with leadership can enhance satisfaction, resilience, and cohesion, ultimately improving care quality.

## Use of AI tools declaration

During the preparation of this manuscript, the authors used Scholar GPT and Scite to identify relevant academic sources, summarize selected papers and to improve grammar, and readability. These tools were used only to assist manuscript preparation and did not contribute to the study design, data collection, statistical analysis, or interpretation of findings. All AI-assisted outputs were critically reviewed, verified, and edited by the authors, who take full responsibility for the accuracy, integrity, and final content of the manuscript.
